# Financial incentives versus standard of care to improve patient compliance with live kidney donor follow-up: protocol for a multi-center, parallel-group randomized controlled trial

**DOI:** 10.1186/s12882-020-02117-9

**Published:** 2020-11-09

**Authors:** Macey L. Levan, Madeleine M. Waldram, Sandra R. DiBrito, Alvin G. Thomas, Fawaz Al Ammary, Shane Ottman, Jaclyn Bannon, Daniel C. Brennan, Allan B. Massie, Joseph Scalea, Rolf N. Barth, Dorry L. Segev, Jacqueline M. Garonzik-Wang

**Affiliations:** 1 Department of Surgery, Division of Transplantation, Johns Hopkins University School of Medicine, 2000 E. Monument Street, Baltimore, MD 21205 USA; 2 Department of Acute and Chronic Care, Johns Hopkins School of Nursing, Baltimore, MD USA; 3 Department of Epidemiology, University of North Carolina, Chapel Hill, NC USA; 4 Department of Epidemiology, Johns Hopkins School of Public Health, Baltimore, MD USA; 5 Division of Transplantation, University of Maryland School of Medicine, Baltimore, MD USA

**Keywords:** Motivation, Financial incentive, Kidney transplantation, Organ donors, Care management, Follow-up, Randomized control trial, Quality improvement, health care, Patient care management, Protocol

## Abstract

**Background:**

Live kidney donors (LKDs) account for nearly a third of kidney transplants in the United States. While donor nephrectomy poses minimal post-surgical risk, LKDs face an elevated adjusted risk of developing chronic diseases such as hypertension, diabetes, and end-stage renal disease. Routine screening presents an opportunity for the early detection and management of chronic conditions. Transplant hospital reporting requirements mandate the submission of laboratory and clinical data at 6-months, 1-year, and 2-years after kidney donation, but less than 50% of hospitals are able to comply. Strategies to increase patient engagement in follow-up efforts while minimizing administrative burden are needed. We seek to evaluate the effectiveness of using small financial incentives to promote patient compliance with LKD follow-up.

**Methods/design:**

We are conducting a two-arm randomized controlled trial (RCT) of patients who undergo live donor nephrectomy at The Johns Hopkins Hospital Comprehensive Transplant Center (MDJH) and the University of Maryland Medical Center Transplant Center (MDUM). Eligible donors will be recruited in-person at their first post-surgical clinic visit or over the phone. We will use block randomization to assign LKDs to the intervention ($25 gift card at each follow-up visit) or control arm (current standard of care). Follow-up compliance will be tracked over time. The primary outcome will be complete (all components addressed) and timely (60 days before or after expected visit date), submission of LKD follow-up data at required 6-month, 1-year, and 2-year time points. The secondary outcome will be transplant hospital-level compliance with federal reporting requirements at each visit. Rates will be compared between the two arms following the intention-to-treat principle.

**Discussion:**

Small financial incentivization might increase patient compliance in the context of LKD follow-up, without placing undue administrative burden on transplant providers. The findings of this RCT will inform potential center- and national-level initiatives to provide all LKDs with small financial incentives to promote engagement with post-donation monitoring efforts.

**Trial registration:**

ClinicalTrials.gov number: NCT03090646

Date of registration: March 2, 2017

Sponsors: Johns Hopkins University, University of Maryland Medical Center

Funding: The Living Legacy Foundation of Maryland

**Supplementary Information:**

The online version contains supplementary material available at 10.1186/s12882-020-02117-9.

## Background

Live donor kidney transplantation is the optimal treatment modality for patients with end-stage renal disease (ESRD), with superior clinical outcomes and quality of life compared to deceased donor kidney transplantation or remaining on dialysis [1–4]. Live kidney donors currently contribute nearly one third of the kidneys used for transplantation each year in the US [5].. While live kidney donation poses minimal post-surgical risks to patients [6–8], donors face a small but measurable increase in the risk of developing ESRD and other chronic diseases in the long-term [9–15]. Prior work suggests that donors who develop de novo disease are at greater risk for ESRD [16, 17]. Hypertension, diabetes mellitus, and glomerulonephritis account for over 60% of ESRD cases in donors [17]. Routine laboratory and clinical screening can detect subclinical disease indicators such as hyperglycemia, elevated blood pressure, proteinuria, and hematuria long before they progress to chronic disease or ESRD. In fact, our group’s preliminary work found that serum creatinine measured as early as 6 months postdonation was predictive of later ESRD development; a 10 mL/min/1.73m^2^ increase in postdonation eGFR was associated with 40% decreased risk of ESRD [18].

Transplant hospitals are required to monitor donors for 2 years postdonation but have historically struggled to meet federally-mandated data reporting requirements. For donors from 2008 to 2012, the vast majority of transplant hospitals had noncompliant 2-year follow-up data; 69 and 84% of hospitals were noncompliant with clinical and laboratory data reporting requirements, respectively [19]. In 2013, the Organ Procurement and Transplantation Network (OPTN) implemented a policy mandating that hospitals report clinical data (i.e. presence of hypertension, diabetes, dialysis, kidney related complications, recent hospitalizations, medical insurance status, income, and vital status) for 80% and laboratory data (i.e. serum creatinine and urine protein) for 75% of donors at 6-months, 1-year, and 2-years postdonation (Supplement 1). While the rate of compliant donor follow-up increased after this policy change (33% pre-policy to 54% post-policy), only 43% of transplant hospitals met all OPTN-mandated 6-month, 1-year, and 2-year thresholds for donors who donated in 2013 [20]. Transplant hospitals commonly cite barriers such as donor inconvenience, direct and indirect costs to donors, donors not wanting to return to the program, and the burden of data collection [21, 22]. Tools to improve donor engagement and strategies that mitigate patient and administrative burden are needed.

Financial incentives have been employed in many realms of healthcare to change health-related behaviors. Financial incentives include a variety of rewards that have an economic value for the recipient, including cash payments, coupons, goods, and services, and have been shown to positively influence both simple (i.e. accomplished through a single action) and complex (i.e. accomplished repeatedly over a period of time, often involving sustained lifestyle modifications) health-related behaviors [23]. Several randomized controlled trials (RCTs) and reviews have found financial incentivization to be associated with increased uptake of recommended preventive services, including smoking abstinence, vaccination, and preventive colonoscopy and mammogram screenings [23, 24]. For example, one RCT found that a $100 incentive more than doubled rates of employee participation in preventive colonoscopy screenings [25]. However, other RCTs have shown a benefit only in subgroups of patients who were at higher risk for poor adherence at baseline [26], while still others observed no difference in outcomes between a financial incentive intervention and control groups [23, 27]. Prior work suggests that the effectiveness of financial incentives in achieving health behavior often varies based on the characteristics of the population and health behavior of interest and may decrease over time [28–31]. Moreover, much of the published literature examining the use of financial incentivization to modify health behavior focuses on low-income populations [28].

Given that patient-level factors are commonly cited by transplant hospitals as barriers to compliance with federally-mandated donor follow-up thresholds [21, 22], financial incentives might be a valuable tool to promote patient engagement in postdonation monitoring efforts. However, given the uncertainty in the literature, an RCT is necessary to evaluate the effectiveness of using financial incentivization to promote patient compliance with follow-up care in this setting.

## Methods

### Objective

To evaluate the effectiveness of financial incentivization in increasing rates of compliance with postdonation follow-up among live kidney donors at two US transplant hospitals.

### Study design

**Fig. 1:**
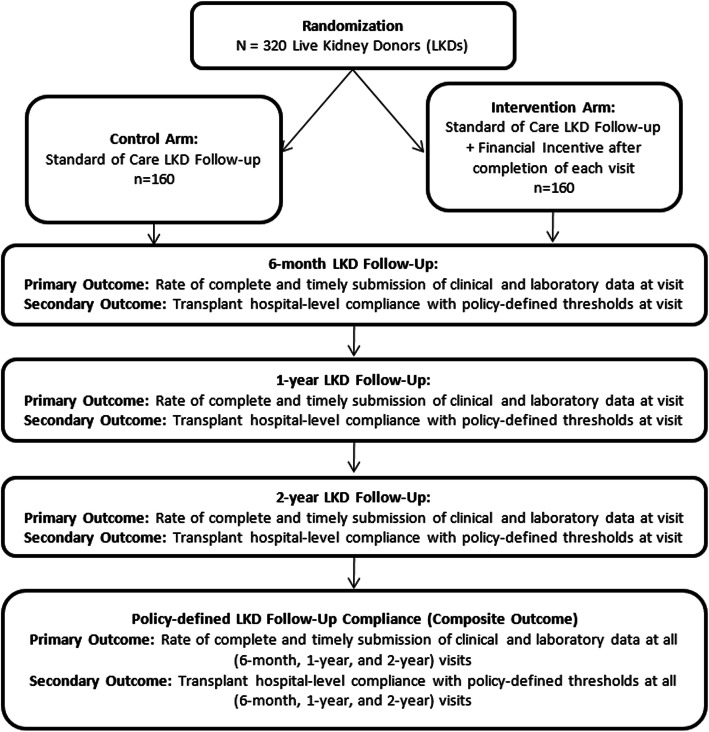
Schematic of Study Design

This trial is designed as a randomized, controlled, nonblinded, two-arm, superiority trial with a 1:1 allocation ratio (NCT03090646). Patients who undergo donor nephrectomy at the Johns Hopkins Hospital Comprehensive Transplant Center (MDJH) and the University of Maryland Medical Center Transplant Center (MDUM) will be eligible for participation. The planned study recruitment period will be 4 years for MDJH and 3 years for MDUM. After consent and randomization, participants will be followed for the federally-mandated 2-year follow-up period (Fig. 1). The primary outcome will be the rate of policy-defined complete (all components addressed) and timely (within 60 days before or after the 6-month, 1-year, or 2-year postdonation date; i.e. 120-day period) submission of data at all 6-month, 1-year, and 2-year follow-up visits. The secondary outcome will be transplant hospital-level compliance with OPTN reporting requirements (submission of clinical data for 80% and laboratory data for 75% of donors) at each visit. Outcomes will be assessed separately for each follow-up time point and as a composite outcome over the study period, and will be compared between study arms following the intention-to-treat principle. We will also collect and report data related to potential logistical challenges of implementing the intervention (i.e., number of mailing attempts necessary, failed delivery attempts, incorrect or out-of-date contact information, etc.).

#### Interventions

The Johns Hopkins Institutional Review Board (IRB) prefers using gift cards rather than cash compensation for clinical research. We chose to provide gift cards to Amazon.com because this online retailer is broadly accessible, and they have been used for other studies within our research group. The gift card will be mailed to participants assigned to the intervention arm after complete (i.e. all components addressed) and timely (i.e. within the 120-day period) submission of follow-up data at each 6-month, 1-year, and 2-year follow-up visit, for a total maximum financial incentive of $75. Each patient will have a 50% chance to be in the intervention arm of the study.

Per MDJH and MDUM policy, a follow-up visit can include either (1) attending an outpatient clinic visit at which the required clinical and laboratory data are collected or (2) submitting a questionnaire documenting a remote standard of care clinic visit and required laboratory values. Participants assigned to the intervention and control arms will receive the same care from the transplant hospital. In other words, those assigned to the control arm will be required to complete the same follow-up activities as those in the intervention arm (per standard of care), but will not receive a gift card. In the United States, donors receive no compensation for postdonation follow-up activities, so the intervention is the only financial incentive provided to donors.

#### Allocation sequence and assignment

Participants will be randomly assigned to either control or experimental group with a 1:1 allocation as per a computer-generated randomization schedule using block randomization with random block sizes ranging from 2 to 8. Block randomization will improve the probability of having balanced groups over the course of the study and during shorter time horizons. A research data analyst on the MDJH study team, blind to the group allocations, will use Stata 15/MP for Linux (College Station, TX, USA) to generate a list of sequential group assignments. This list will be used to create sequentially numbered, sealed, opaque envelopes that will be used to allocate consenting participants to the control or intervention arms of our study. Study personnel who create the sealed envelopes will not be involved in patient recruitment. Study personnel who conduct recruitment will not be permitted to view the list of sequential group assignment during the study period, and the envelope will not be opened until after a patient has provided informed consent to participate in the study; therefore, the study group allocation will be concealed to both participants and recruiters until after study enrollment.

Patients, healthcare workers on the study team, and study team members responsible for data collection and analysis will be aware of which arm participants are randomized to. Therefore, this study will not be blinded to providers, patients, or study personnel, but will be blinded to data analysts.

### Study population

We will recruit donors for study participation at two urban US transplant programs affiliated with large, academic hospitals. Both MDJH and MDUM have an average annual volume of approximately 80 donors per year, and are considered large-volume transplant hospitals.

We plan to enroll 320 participants during the study period. Donors randomized to the intervention arm (approx. *N* = 160) will receive a gift card upon completion of each follow-up visit. Donors randomized to the control arm (approx. *N* = 160) will receive the standard of care for donor follow-up at MDJH and MDUM.

#### Inclusion and exclusion criteria

Donors who undergo nephrectomy at MDJH and MDUM in the study period will be eligible for participation in this RCT. We will exclude only donors who do not speak English, do not live in the US, or do not consent to participate in the study. Per national policy, all living donors are ≥18 years of age.

### Study procedures

Study personnel at each site will be responsible for approaching patients who undergo donor nephrectomy at their respective sites (i.e. MDJH study personnel will recruit MDJH patients and MDUM study personnel will recruit MDUM patients). At each site, study personnel will obtain a list of patients who have undergone donor nephrectomy from surgical providers (co-investigators on this study) or hospital records and will approach all of these patients as possible participants. Only requisite study personnel will have access to lists of potential patients. Study personnel will approach donors in one of the following settings: (1) the inpatient clinical transplant unit after patients have undergone donor nephrectomy, prior to their discharge; or (2) at the donor’s first medically-required outpatient clinic visit. Informed consent will be documented using a written consent form. If patients are unable to be contacted at these times, study personnel will contact the patient via telephone and describe the study using a telephone screening script. If patients are not interested in learning more about the study, study personnel will record this refusal. If patients are interested in participating, study personnel will use an oral consent script to obtain consent. The number of acceptances, eligible enrollments, and refusals will be recorded; then the list will be destroyed. Surgeon and clinician members of the study team will not participate in recruitment activities to avoid the potential for coercion and appearance of conflict of interest. As much time as necessary will be allowed for obtaining consent. After providing informed consent, participants will be randomized to the intervention or the control arms using opaque, sealed envelopes.

All study participants will be instructed to complete required follow-up activities as is standard of care. Study personnel at MDJH and MDUM will monitor patient compliance with 6-month, 1-year, and 2-year follow-up. Study personnel at MDUM will provide periodic enrollment and follow-up compliance updates to the MDJH study team using a HIPAA-compliant REDCap database [32]. Enrollment data will include patient name, date of birth, date of donation, demographic information (including gender, race, and ethnicity), contact information, and an electronic copy of the informed consent form. Follow-up compliance data will include the dates of completion of clinical (i.e. questionnaire) and laboratory follow-up components for each participant. Only requisite study personnel will have access to the REDCap database.

The MDJH study team will mail gift cards to participants enrolled at both MDJH and MDUM who were assigned to the intervention arm. Gift cards will be mailed with a letter reminding participants that the gift card is for their complete and timely submission of required donor follow-up activities. Gift cards will be mailed using USPS Domestic Certified Mail, which requires the recipient to sign for the letter at the time of delivery and provides the sender with a mailing receipt and electronic verification of delivery. The number of mailing attempts and dates of gift card mailing and receipt by the participant will be recorded.

There will not be study-specific efforts to retain participants, as this would be a form of intervention that might impact outcomes. However, transplant providers at MDJH or MDUM may contact donors for the purpose of obtaining complete and timely follow-up data in order to comply with nationally-mandated follow-up requirements. The hospital protocols for continued patient follow-up are consistent between MDJH and MDUM. Participants may withdraw from the RCT at any time without penalty. Withdrawal from the RCT would not preclude participants from obtaining regular medical care or follow-up care related to their kidney donation. If participants choose to withdraw, the study team will use the data collected prior to withdrawal and mark the remaining data as censored. Other than interventions that might impact rates of donor follow-up compliance, no concomitant care or interventions will be prohibited during the trial.

#### Data collection

The enrollment data for MDJH and MDUM will be added by site personnel to a HIPAA-compliant REDCap database. We will collect the name, birth date, donation date, demographic information (gender, race, ethnicity), and contact information for every patient who consents to participate. We will also collect follow-up compliance data, including the dates of completion of clinical (questionnaire) and laboratory follow-up components for each participant. For participants in the intervention arm, we also record the dates that each gift card was mailed and received, and if there was a failed delivery of the gift card via the USPS Certified Mail tracking system.

### Statistical analysis

Rates of patient and center-level compliance for each 6-month, 1-year, and 2-year follow-up visit will be compared between study arms using Fischer’s exact test. Rates of patient and center-level compliance for the composite outcome of compliant 6-month, 1-year, AND 2-year follow-up visit will be compared between study arms using Pearson’s chi-squared test. All analyses will follow an intent-to-treat principle. Data will be analyzed by data analysts on the MDJH study team using Stata 15/MP for Linux (College Station, Texas).

Our primary end-points are the living donor policy defined outcomes of complete and timely 6-month, 1-year, and 2-year follow-up. The motivation for these outcomes is to align our findings with the relevant policy end-points, not to maximize statistical power. As an additional secondary aim, generalized estimating equations (GEE) will be used to estimate population-averaged changes in complete and timely follow-up over the full course of the study. This approach will allow for participants with non-policy compliant follow-up behaviors (e.g. missing 1-year follow-up but completing 6- and 2-year follow-up) to be counted towards a population average. This may provide insight on alternative designs for the living donor follow-up policy.

#### Sample size calculation

**Fig. 2:**
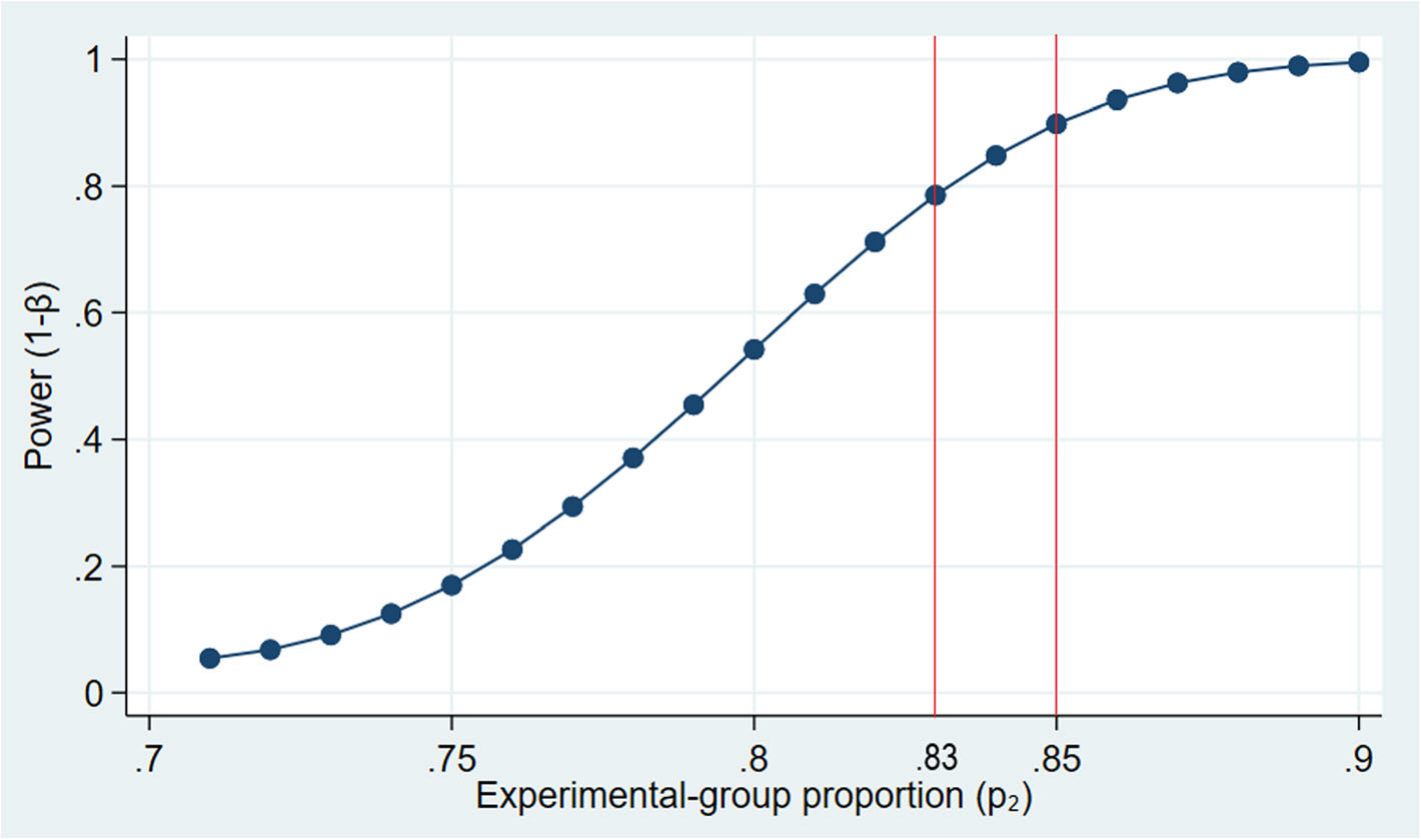
Power calculation of evaluating the effectiveness of the financial incentive intervention with 320 donors enrolled at MDJH and MDUM over 4 years

The total sample size for this study (including participants at both MDJH and MDUM) is *N* = 320. With 160 participants in each arm given the hypothesized follow-up in control group of 70%, we will have 80% power to detect a difference between the study arms if the follow-up rate is 83% in the intervention group, and 90% power to detect a difference between study arms if follow-up in intervention group is 85% (Fig. 2). The hypothesized follow-up in the control group is derived from historical follow-up percentages at MDJH and MDUM.

### Ethics and dissemination

#### Research ethics approval

This study was approved by the Johns Hopkins School of Medicine IRB (IRB00126158) and the University of Maryland Medical Center IRB (HP-00081637). Amendments to the protocol will be submitted to the Johns Hopkins School of Medicine and University of Maryland Medical Center IRBs for review and approval. Subsequent to initial review and approval, the responsible local IRBs will review the protocol at least annually. Participants will be notified of any significant changes to the study design via a mailed letter using the information collected at the time of recruitment. The clinical and research activities being reported are consistent with the Declaration of Helsinki and Declaration of Istanbul.

#### Participant and data confidentiality

Only requisite study personnel at MDJH and MDUM will have access to identifying patient information. Johns Hopkins study team members will receive data about whether patients enrolled in the RCT at MDUM completed their required 6-month, 1-year, and 2-year follow-up visits but will have no direct patient contact with these donors. Study data will be collected and managed using REDCap electronic data capture tools hosted at Johns Hopkins University [32]. Data will be stored on the REDCap server for 7 years according to HIPAA requirements. All study personnel have received requisite training in data confidentiality and human subjects research.

#### Data safety and trial monitoring

The Johns Hopkins School of Medicine IRB determined that a data monitoring committee was not necessary for this RCT due to minimal participant risk. Data monitoring will be conducted and reported by the PI as projected by the data safety monitoring plan. The PI will immediately report any unanticipated adverse events or study deviations to the Johns Hopkins School of Medicine IRB.

#### Dissemination plan

Summary results of this RCT will be reported to ClinicalTrials.gov no later than 1 year after the study completion date, as per the NIH Policy on Dissemination of NIH-Funded Clinical Trial Information [33]. We also anticipate submitting the findings of this pilot RCT for peer-reviewed publication. Authorship eligibility will be determined using ICJME guidelines [34].

## Discussion

This randomized controlled trial will provide valuable information about whether using small financial incentives is an effective strategy to promote patient compliance with donor follow-up. If financial incentivization proves to be effective in this setting, the results of this RCT could serve as evidence to guide potential center-level and national initiatives to provide financial incentives for all live kidney donors. On the other hand, if financial incentivization does not effectively improve compliance, this will suggest that alternative interventions to increase live donor engagement with post-donation follow-up care should be explored.

This RCT has several limitations which merit discussion. Historically, donors have been predominantly Caucasian [5, 35]. In 2016, Caucasian donors donated 70% of living donor kidneys in the United States [5]; comparatively, 77% of the donors in our cohort are Caucasian. As such, our results may have limited generalizability to minority populations (ex: African American, Hispanic, and Asian donors). In addition, because the gift cards are mailed to participants assigned to the intervention arm rather than distributed in person, it is possible that some may not reach the intended recipient. However, we will use USPS Certified Mail, which requires a signature by the recipient for delivery, and will record any delivery failures. Finally, it is possible that unblinded intervention arm assignment could result in ascertainment bias; however, it would be infeasible to blind study participants in this RCT, as the intervention is dependent on the participant’s expectation of a financial incentive for follow-up completion.

Despite the aforementioned limitations, this trial has several key strengths. To our knowledge, it is the first clinical trial to assess the effectiveness of using financial incentives to promote donor compliance with postdonation monitoring efforts by a transplant hospital. Additionally, our randomized design will provide high-quality evidence to inform center-level and national efforts to improve donor follow-up compliance. If found to be effective, financial incentivization could serve as a useful strategy in center-level and national initiatives to improve postdonation care for live kidney donors.

## Supplementary Information

**Additional file 1.** : Supplement 1. UNOS Living Donor Follow-up Worksheet. This file contains the information transplant centers are required to collect and report on living donors at each follow-up timepoint.

## Data Availability

Not applicable.

## Abbreviations

IRB: Institutional review board
MDJH: Johns Hopkins Hospital Comprehensive Transplant Center
MDUM: University of Maryland Medical Center Transplant Center
RCT: Randomized controlled trial
LDKT: Live donor kidney transplantation
ESRD: End-stage renal disease
OPTN: Organ Procurement and Transplantation Network
