# Supplementary material for: Financial incentives versus standard of care to improve patient compliance with live kidney donor follow-up: protocol for a multi-center, parallel-group randomized controlled trial
Source: BMC Nephrol. 2020 Nov 9;21:465. doi: 10.1186/s12882-020-02117-9 (PMC7654057; doi:10.1186/s12882-020-02117-9)
Supplement: Supplementary file 1 — Additional file 1. : Supplement 1. UNOS Living Donor Follow-up Worksheet. This file contains the information transplant centers are required to collect and report on living donors at each follow-up timepoint. [file 12882_2020_2117_MOESM1_ESM.pdf]

Records

Living Donor Follow-Up Worksheet

FORM APPROVED: O.M.B. NO. 0915-0157 Expiration Date: 07/31/2020

Note: These worksheets are provided to function as a guide to what data will be required in the online TIEDI® application. Currently in the worksheet, a red asterisk is displayed by fields that are required, independent of what other data may be provided. Based on data provided through the online TIEDI® application, additional fields that are dependent on responses provided in these required fields may become required as well. However, since those fields are not required in every case, they are not marked with a red asterisk.

Donor ID:

Provider Information

Recipient Center:

Followup Center:

Donor Information

Name: **DOB:**

Transplant Date:

SSN: **Gender:**

Donor ID: **Recovery Date:**

Organ:

Donor Status

Date of Initial Discharge:

Date of last contact or death: *

Most Recent Donor Status since: * Living

Attempts to Collect: *

Cause of Death:

Specify:

Functional Status:

Physical Capacity: *
- ○ No Limitations
- ○ Limited Mobility
- ○ Wheelchair bound or more limited
- ○ Unknown

Working for Income: * ○ YES ○ NO ○ UNK

If No, Not Working Due To:
- ○ Disability
- ○ Insurance Conflict
- ○ Inability to Find Work
- ○ Donor Choice - Homemaker
- ○ Donor Choice - Student Full Time/Part Time
- ○ Donor Choice - Retired
- ○ Donor Choice - Other
- ○ Unknown

If Yes:
- ○ Working Full Time
- ○ Working Part Time due to Disability
- ○ Working Part Time due to Insurance Conflict
- ○ Working Part Time due to Inability to Find Full Time Work
- ○ Working Part Time due to Donor Choice
- ○ Working Part Time Reason Unknown
- ○ Working, Part Time vs. Full Time Unknown

Loss of Insurance Due to Donation: ○ YES ○ NO ○ UNK

If Yes:
- ☐ Loss of Health Insurance
- ☐ Loss of Life Insurance

Clinical Information

Current weight: * **Date:** ____ ____ lb ____ kg **ST=** ____

ER or urgent care visit related to donation since last follow-up: * ○ YES ○ NO ○ UNK

Liver Clinical Information

Most Recent Values Since:

Total Bilirubin: **Date:**

			mg/dl	ST=
**SGOT/AST:**	**Date:**		U/L	ST=
**SGPT/ALT:**	**Date:**		U/L	ST=
**Alkaline Phosphatase:**	**Date:**		units/L	ST=
**Serum Albumin:**	**Date:**		g/dl	ST=
**Serum Creatinine:**	**Date:**		mg/dl	ST=
**INR:**	**Date:**			ST=
**Platelet count:**	**Date:**		mL	ST=

Kidney Clinical Information

Most Recent Values Since:

**Serum Creatinine:**	**Date:**		mg/dl	ST=
**Blood Pressure Systolic:**	**Date:**		mm/Hg	ST=
**Blood Pressure Diastolic:**	**Date:**		mm/Hg	ST=

Donor Developed Hypertension Requiring Medication: YES NO UNK

Urinalysis:

Urine Protein:
- Positive
- Negative
- Not Done
- Unknown

or

Protein-Creatinine Ratio: GM/GM

Maintenance Dialysis: YES NO UNK

If Yes, Date First Dialyzed:

Diabetes: YES NO UNK

Treatment:
- Insulin
- Oral Hypoglycemic Agent
- Diet

Lung Clinical Information

Activity Level:
- No change in activity level
- Mild decrease in activity level
- Moderate decrease in activity level
- Severe decrease in activity level
- Increase in activity level
- Unknown

Chronic Incisional Pain:
- Mild
- Moderate
- Severe
- Unknown

Complications

Has the donor been readmitted since: YES NO UNK

If Yes, Date of First Readmission: ST=

Specify Reason for First Readmission:

Kidney Complications since: YES NO UNK

If Yes:
- Added to UNOS TX candidate waiting list
- Other, specify

Specify:

Liver Complications since: YES NO UNK

If Yes:
- Bile Leak
- Hepatic Resection
- Abscess

- [ ] **Liver Failure**
- [ ] **Added to UNOS TX candidate waiting list**
- [ ] **Incisional hernia due to donation surgery**
- [ ] **Other, specify**

Specify:

Complications since: ◯ **YES** ◯ **NO**

Specify:

Recipient Information

Name:

Transplant Date:

SSN:

Copyright © 2018 United Network for Organ Sharing. All rights reserved.

Confidential - OPTN Use Only
